# Frontline brentuximab vedotin-based therapy for newly diagnosed classical Hodgkin lymphoma: a meta-analysis of randomized controlled trials

**DOI:** 10.3389/fonc.2025.1636923

**Published:** 2025-07-21

**Authors:** Yue Yang, Shu-rong Liu, Li Wang, Rui Zhang, Jing Xie

**Affiliations:** ^1^ Department of Trauma and Hand and Foot Microsurgery, Sunshine Union Hospital, Weifang, China; ^2^ Department of Oncology, Sunshine Union Hospital, Weifang, China; ^3^ Department of Emergency Medicine, Rocket Force Characteristic Medical Center, Beijing, China; ^4^ Department of Gastroenterology, Rocket Force Characteristic Medical Center, Beijing, China

**Keywords:** Hodgkin lymphoma, brentuximab vedotin, meta-analysis, frontline therapy, progression-free survival, safety, randomized controlled trial

## Abstract

**Background:**

Brentuximab vedotin (BV), an anti-CD30 antibody-drug conjugate, has established efficacy in relapsed/refractory Hodgkin lymphoma (HL), yet its role as frontline therapy for newly diagnosed classical HL requires systematic evaluation.

**Methods:**

We conducted a meta-analysis of randomized controlled trials (RCTs) compared BV-based versus conventional non-BV-containing regimens (namely, classical chemotherapeutic regimens) in previously untreated classical HL patients, assessing progression-free survival (PFS), PET metabolic responses (interim PET-2 negativity and end-of-treatment complete response), and safety profiles (grade ≥3 adverse events [AEs], including febrile neutropenia, peripheral neuropathy, and secondary malignancies).

**Results:**

Pooled data from four RCTs (N = 3591 patients) demonstrated significant PFS improvement with BV-based regimens (HR: 0.58, 95% CI: 0.44 to 0.77, *P* < 0.001), with consistent benefits across subgroups stratified by disease stage, gender, age, and International Prognostic Score (IPS). Although interim PET-2 negativity rates showed only a non-significant trend favoring BV (RR: 1.02, 95% CI: 0.99 to 1.04, *P* = 0.286), end-of-treatment complete metabolic response rates were significantly higher (RR: 1.03, 95% CI: 1.00 to 1.06, *P* = 0.024). Safety analyses revealed comparable incidences of grade ≥3 AEs between groups (RR: 1.05, 95% CI: 0.80 to 1.37, *P* = 0.739), with no increased risk of peripheral neuropathy or secondary malignancies.

**Conclusions:**

Our meta-analysis demonstrates that incorporation of BV into frontline therapy for classical HL provides significant PFS benefits and improved end-of-treatment metabolic responses, with manageable toxicity. These findings support BV-based regimens as a promising frontline therapeutic strategy in classical HL, though extended follow-up is required to evaluate long-term survival outcomes.

## 1 Introduction

The combination regimen of doxorubicin, bleomycin, vinblastine, and dacarbazine (ABVD) remains the standard first-line therapy for newly diagnosed Hodgkin lymphoma (HL) (1, 2). Nevertheless, approximately 30% of patients with advanced-stage disease develop refractory or relapsed disease following frontline ABVD therapy (3, 4). Positron emission tomography (PET)-guided strategies, including treatment escalation from ABVD to bleomycin, etoposide, doxorubicin, cyclophosphamide, vincristine, procarbazine, and prednisone (BEACOPP), have demonstrated improved tumor control and survival advantages in advanced-stage HL (5, 6). However, these intensified chemotherapy protocols carry substantially higher risks of severe treatment-related toxicities, particularly secondary malignancies (6–8). There exists an urgent clinical need to develop novel therapeutic strategies that can simultaneously enhance treatment efficacy while reducing toxicity profiles in HL management.

Brentuximab vedotin (BV), an anti-CD30 antibody-drug conjugate (9), has demonstrated clinically meaningful single-agent activity in patients with refractory or relapsed classical HL (10–12). The integration of BV into established HL treatment backbones represents a viable strategy to preserve the robust efficacy of multi-agent chemotherapy while reducing treatment-associated toxicities (13–16). Clinical evidence shows that BV combined with AVD (doxorubicin, vinblastine, dacarbazine) yields superior survival outcomes compared to ABVD, along with significantly reduced pulmonary toxicity in advanced HL patients (17, 18). Furthermore, the BrECADD regimen (BV plus etoposide, cyclophosphamide, doxorubicin, dacarbazine, dexamethasone) has shown enhanced efficacy versus escalated BEACOPP, coupled with improved tolerability profiles in advanced HL patients (19).

However, current evidence shows inconsistent results regarding the efficacy and safety of BV-based regimens in HL patients. The BREACH trial demonstrated that BV-based regimens significantly increased the rate of negative PET response after two cycles of chemotherapy (PET-2) (20). In contrast, the AHOD1331 trial found comparable PET-2 negative rates between BV-based and conventional non-BV-containing treatment groups (21). Additionally, the safety profiles of BV-based regimens require further characterization, as current adverse effects (AEs) reporting may be limited by relatively low event rates.

Therefore, we performed this meta-analysis of randomized controlled trials (RCTs) to systematically assess both the therapeutic efficacy and safety profile of BV-based regimens as first-line treatment for newly diagnosed classical HL patients.

## 2 Materials and methods

### 2.1 Search strategy

A comprehensive literature search was performed in PubMed, the Excerpta Medica Database (Embase), and the Cochrane Central Register of Controlled Trials (CENTRAL) using search items including “brentuximab vedotin,” “Hodgkin lymphoma,” “Hodgkin’s lymphoma,” “Hodgkin disease,” and “Hodgkin’s disease.” The literature search, limited to English-language publications, covered records up to March 2025. The detailed search strategies are listed in **Supplementary Tables 1**-**3**. We identified RCTs comparing BV-based regimens with conventional non-BV-containing regimens (including but not limited to ABVD, BEACOPP, and their variants) in newly diagnosed HL patients. Additional studies were located through manual reference screening of retrieved articles. This meta-analysis was conducted in accordance with the Preferred Reporting Items for Systematic Reviews and Meta-Analyses (PRISMA) guidelines (22).

### 2.2 Selection criteria

Two researchers independently assessed all potentially eligible studies. The primary efficacy endpoints for this meta-analysis included: (1) progression-free survival (PFS), defined as the duration from randomization to first documented relapse, disease progression, or death from any cause; (2) PET response rate after two cycles of treatment (PET-2); and (3) PET response at the end of treatment (PET-EOT). PET-2 and PET-EOT assessments employed the Deauville scoring system (23), with negative PET-2 defined as scores 1–3 and positive PET-2 as scores 4-5. PET complete response at the end of treatment required Deauville scores of 1 or 2. Safety evaluations focused on grade 3–4 AEs, including febrile neutropenia, leukopenia, infections, peripheral neuropathy, and secondary malignancies, graded per Common Terminology Criteria for Adverse Events (version 4.0). We included RCTs comparing BV-based regimens versus conventional non-BV-containing regimens in patients with newly diagnosed HL that reported at least one predefined outcome. Conference abstracts were excluded from analysis. All discrepancies were resolved by discussion.

### 2.3 Data extraction and methodological quality evaluation

Two researchers independently extracted the data. All relevant data were collected, including: (1) reference details (trial name, publication year, and study design); (2) patient characteristics (age, gender, diagnosis, disease stage, and sample size); (3) BV-based regimens (BV doses, frequencies, and treatment cycles); (4) conventional non-BV-containing regimens, and (5) the aforementioned efficacy and safety outcomes. All discrepancies were resolved through discussion. The corresponding authors of included studies were contacted if necessary.

The methodological quality of included studies was evaluated using the Cochrane Collaboration Reviews’ Handbook (24). Specifically, two researchers independently assigned ratings of “low risk,” “high risk,” or “unclear risk” of bias to the following seven items: randomization adequacy, allocation concealment, participant blinding, outcome assessor blinding, incomplete outcome data, selective reporting, and other potential biases.

### 2.4 Statistical analyses

All statistical analyses were conducted with Stata 12.0 (Stata Corporation, College Station, TX, USA). For time-to-event outcomes, we synthesized hazard ratios (HRs) with 95% confidence intervals (CIs). Dichotomous outcomes were analyzed using risk ratios (RRs) with 95% CIs. Study heterogeneity was quantified through the *I^2^* statistic, and *I^2^* >50% and *P* < 0.10 indicated substantial heterogeneity (25). To address potential heterogeneity sources, we performed subgroup or sensitivity analyses where feasible. Regardless of heterogeneity levels, we employed random-effects models for all meta-analyses to ensure conservative estimates (26). Statistical significance was defined as *P* < 0.05 for all analyses.

## 3 Results

### 3.1 Study selection and characteristics

**Figure 1:**
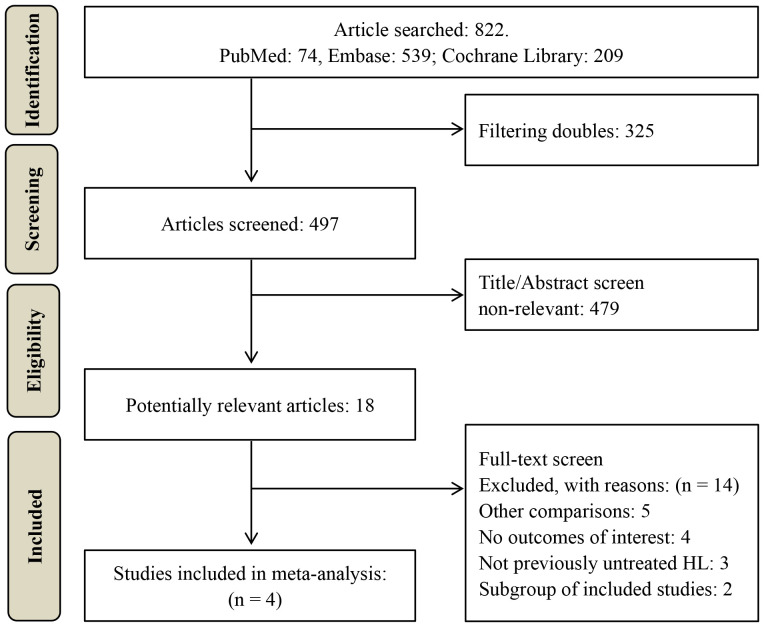
Study selection flow diagram.

A total of 822 potentially relevant studies were identified through database searching. 497 studies remained after duplicate removal. Subsequently, 479 studies were excluded after title and abstract screening. The remaining 18 studies underwent full-text review, resulting in the exclusion of 14 studies. The excluded studies and their respective reasons for exclusion are detailed in **Supplementary Table 4**. Ultimately, four RCTs comprising 3591 classical HL patients (1826 in the BV-based group and 1765 in the conventional non-BV-containing group) were included in this meta-analysis (18–21), as illustrated in **Figure 1**.

The characteristics of the included studies are summarized in **Tables 1** and **2**. The sample size of the included RCTs ranged from 170 to 1500. All four multicenter RCTs (18–21) consistently substituted bleomycin with BV in their experimental arms (18–21). The therapeutic comparisons comprised: two trials evaluating BV-AVD versus ABVD (18, 20); one trial investigating BrECADD versus eBEACOPP (19); and one trial examining BV-AVE-PC versus ABVE-PC (21). Two trials focused exclusively on adult populations (aged ≤60 years) (19, 20). One trial enrolled adults with extended age eligibility (>60 years were also included) (18). One trial specifically assessed pediatric and adolescent populations (21).

**Table 1:** Summary characteristics of the included randomized controlled trials.

References	Publication year	Study design	Phase	Sample size (BV vs control)	BV-based regimens	non-BV-containing regimens	BV benefits
AHOD1331 trial (21)	2022	Multicenter RCT	III	587 (298 vs 289)	Five 21-day cycles of BV (1.8 mg/kg) plus AVE-PC	Five 21-day cycles of ABVE-PC	PFS: yes; PET-2: no; PET-EOT: no
ECHELON-1 trial (18)	2022	Multicenter RCT	III	1334 (664 vs 670)	Six 28-day cycles of BV (1.2 mg/kg, d1, d15) plus AVD	Six 28-day cycles of ABVD	PFS: yes; PET-2: no; PET-EOT: no
BREACH trial (20)	2023	Multicenter RCT	II	170 (113 vs 57)	Four 28-day cycles of BV (1.2 mg/kg, d1, d15) plus AVD	Four 28-day cycles of ABVD	PFS: no; PET-2: yes
GHSG HD21 trial (19)	2024	Multicenter RCT	III	1500 (751 vs 749)	Four or six 21-day cycles of BrECADD (BV 1.8 mg/kg)	Four or six 21-day cycles of eBEACOPP	PFS: yes; PET-2: no; PET-EOT: no

BV, brentuximab vedotin; AVE-PC, doxorubicin, vincristine, etoposide, prednisone, and cyclophosphamide; ABVE-PC, doxorubicin, bleomycin, vincristine, etoposide, prednisone, and cyclophosphamide; AVD, doxorubicin, vincristine, and dacarbazine; ABVD, doxorubicin, bleomycin, vincristine, and dacarbazine; BrECADD, brentuximab vedotin, etoposide, cyclophosphamide, doxorubicin, dacarbazine, and dexamethasone; eBEACOPP, escalated doses of bleomycin, etoposide, doxorubicin, cyclophosphamide, vincristine, procarbazine, and prednisone; PFS, progression-free survival; PET, positron emission tomography; PET-2, PET response rate after two cycles of treatment; PET-EOT, PET complete response rate at the end of treatment.

**Table 2:** Patient characteristics for the included randomized controlled trials.

References	Patients	Age (years)		Gender		Ann Arbor stage		Follow up periods (months)
		BV-based regimens	non-BV-containing regimens	BV-based regimens	non-BV-containing regimens	BV-based regimens	non-BV-containing regimens	
AHOD1331 trial (21)	Newly diagnosed stage IIB with bulk tumor or stage IIIB, or IV HL	15.4 (3.4-22.0)	15.8 (4.6-21.5)	Female: 138 (46.3%)	Female: 138 (47.8%)	IIB with bulk tumor: 62 (20.8%), IIIB: 59 (19.8%), IVA: 84 (28.2%), IVB: 93 (31.2%)	IIB with bulk tumor: 59 (20.4%), IIIB: 54 (18.7%), IVA: 83 (28.7%), IVB: 93 (32.2%)	42.1 (0.1-80.9)
ECHELON-1 trial (18)	Newly diagnosed stage III or IV HL	35 (18–82)	37 (18–83)	Female: 286 (43.1%)	Female: 272 (40.6%)	II: 1 (0.2%), III: 237 (35.7%), IV: 425 (64.0%); NA: 1 (0.2%)	III: 246 (36.7%), IV: 421 (62.8%); NA: 3 (0.4%)	73.0 (0.0-100.6)
BREACH trial (20)	Newly diagnosed early-stage unfavorable HL	29 (18–59)	28 (18–60)	Female: 60 (53.1%)	Female: 26 (45.6%)	I: 8 (7.1%), II: 104 (92.0%), III: 1 (0.9%)	I: 3 (5.3%), II: 53 (93.0%), III: 1 (1.7%)	45.0 (0.2-60.6)
GHSG HD21 trial (19)	Newly diagnosed advanced-stage HL	31 (24–42)	31 (24–42)	Female: 323 (43.5%)	Female: 321 (43.4%)	IIA: 2 (0.3%), IIB: 115 (15.5%), IIIA: 129 (17.4%), IIIB: 164 (22.1%), IVA: 104 (14.0%), IVB: 228 (30.7%)	IIB: 117 (15.8%), IIIA: 132 (17.9%), IIIB: 156 (21.1%), IVA: 112 (15.2%), IVB: 222 (30.0%)	48.0 (NA)

HL, Hodgkin lymphoma; BV, brentuximab vedotin; NA, not available.

Data are presented as n (%) for categorical variables, and as median (range) or median (interquartile range) for age and follow-up duration.

### 3.2 Methodological quality evaluation

All included RCTs (18–21) explicitly described randomization. However, none of these studies implemented allocation concealment (18–21). Given their open-label design, participant blinding was not performed in any trial (18–21); only one study (20) reported blinding of outcome assessors. Regarding other quality indicators, including incomplete outcome data, selective reporting, and other biases, all included studies were judged to have low risk of bias. The complete methodological quality assessment for the included RCTs is presented in **Supplementary Figure 1**.

### 3.3 PFS and subgroup analysis

**Figure 2:**
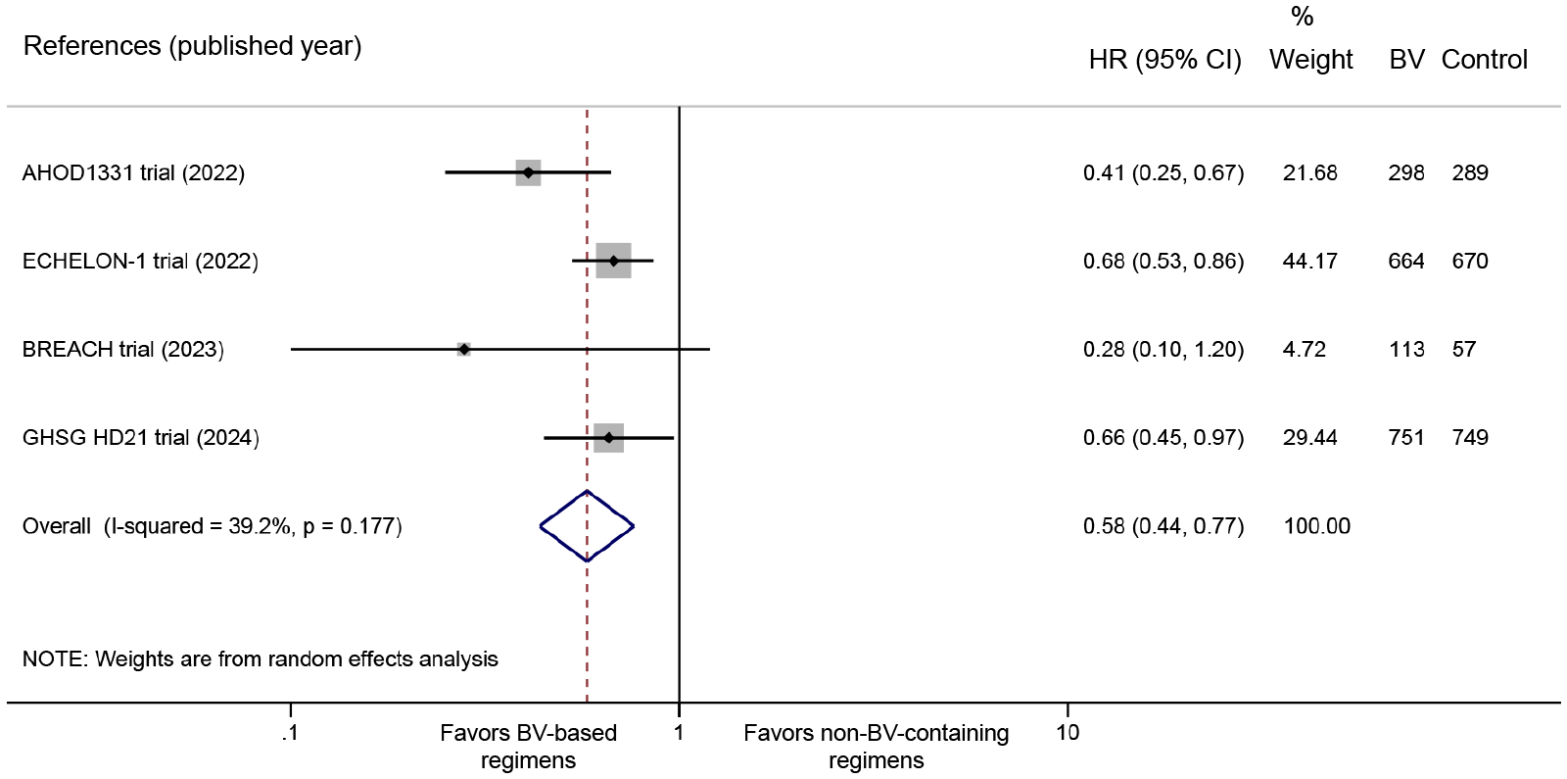
Forest plot of PFS outcomes comparing BV-based regimens versus conventional non-BV-containing regimens in newly diagnosed classical HL patients. PFS, progression-free survival; BV, brentuximab vedotin; HL, Hodgkin lymphoma. HR <1 favors BV-based regimens.

All four RCTs reported PFS data. The pooled analysis demonstrated significantly improved PFS with BV-based regimens compared to conventional non-BV-containing regimens in newly diagnosed classical HL patients (HR: 0.58, 95% CI: 0.44 to 0.77, *P* < 0.001; *I^2^* = 39.2%, *P* = 0.177) (**Figure 2**). Despite the absence of statistical heterogeneity, we performed sensitivity analyses to address clinical heterogeneity, demonstrating robust PFS outcomes (HR range: 0.50 to 0.66) that aligned with our primary analysis (**Supplementary Table 5**).

**Figure 3:**
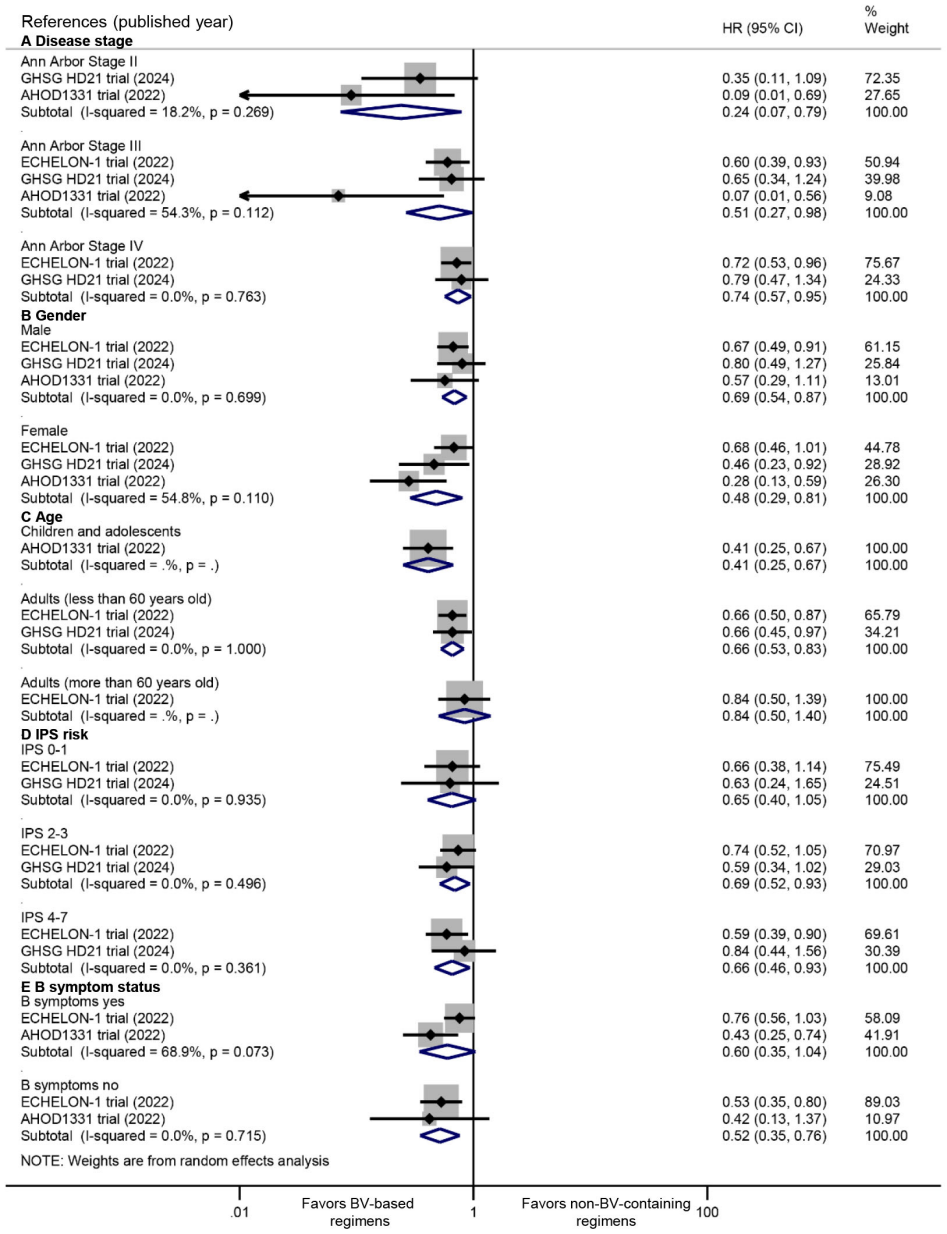
Subgroup analyses of PFS benefits from BV-based regimens stratified by: **(A)** disease stage, **(B)** gender, **(C)** age, **(D)** International Prognostic Score (IPS) risk categories, and **(E)** B symptom status. PFS, progression-free survival; BV, brentuximab vedotin. HR <1 favors BV-based regimens.

Consistent PFS benefits were observed across disease stages (Stage II: HR 0.24, 95% CI: 0.07 to 0.79, *P* = 0.019; Stage III: HR 0.51, 95% CI: 0.27 to 0.98, *P* = 0.043; Stage IV: HR 0.74, 95% CI: 0.57 to 0.95, *P* = 0.020) (**Figure 3A**). Significant PFS improvements were evident in both male (HR 0.69, 95% CI: 0.54 to 0.87, *P* = 0.002) and female patients (HR: 0.48, 95% CI: 0.29 to 0.81, *P* = 0.005) (**Figure 3B**). Significantly favorable PFS benefits were observed in younger populations (children and adolescents: HR 0.41, 95% CI: 0.25 to 0.67, *P* < 0.001; adults <60 years: HR 0.66, 95% CI: 0.53 to 0.83, *P* < 0.001), while not in adults ≥60 years (HR 0.84, 95% CI: 0.50 to 1.40, *P* = 0.504) (**Figure 3C**). Furthermore, the PFS advantage was observed in intermediate/high International Prognostic Score groups (IPS 2-3: HR 0.69, 95% CI: 0.52 to 0.93, *P* = 0.015; IPS 4-7: HR 0.66, 95% CI: 0.46 to 0.93, *P* = 0.018) (**Figure 3D**), and patients without B symptoms (HR 0.52, 95% CI: 0.35 to 0.76, *P* = 0.001) (**Figure 3E**).

### 3.4 PET-2 and PET-EOT evaluation

**Figure 4:**
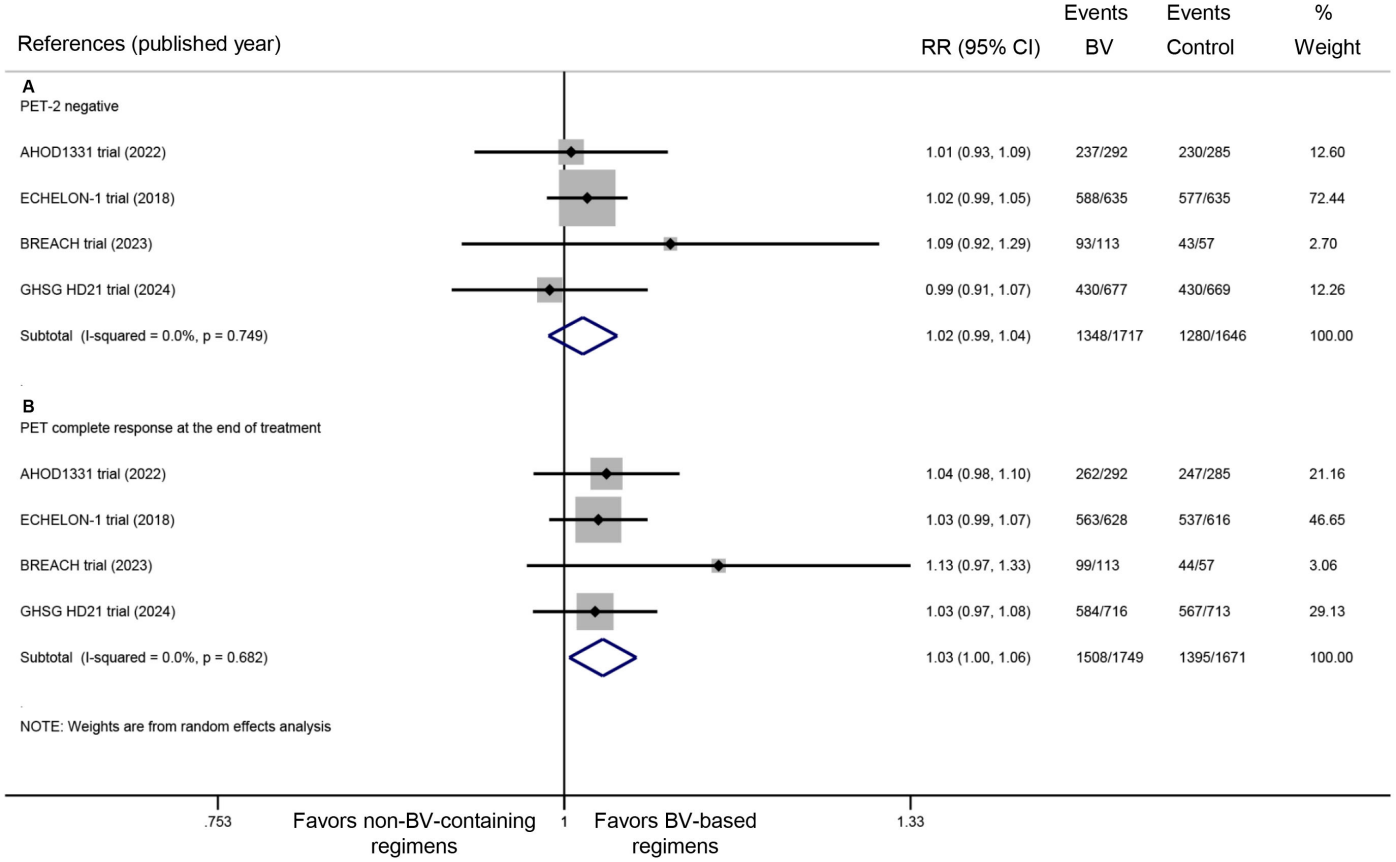
Metabolic response rates with BV-based regimens: **(A)** PET negativity after two cycles of treatment, and **(B)** end-of-treatment complete metabolic response. BV, brentuximab vedotin; PET, positron emission tomography. RR >1 favors BV-based regimens (PET response).

All four included RCTs provided data on PET response rates both after the second treatment cycle and at the end of treatment. Pooled analysis revealed no statistically significant difference in PET-2 negative rates between BV-based and conventional non-BV-containing regimens, though a trend favoring the BV-based group was observed (RR: 1.02, 95% CI: 0.99 to 1.04, *P* = 0.286; *I^2^* = 0.0%, *P* = 0.749) (**Figure 4A**). In contrast, significantly higher rates of complete metabolic response were demonstrated in the BV-based group at end-of-treatment evaluation (RR: 1.03, 95% CI: 1.00 to 1.06, *P* = 0.024; *I^2^* = 0.0%, *P* = 0.682) (**Figure 4B**).

### 3.5 Safety profile

**Figure 5:**
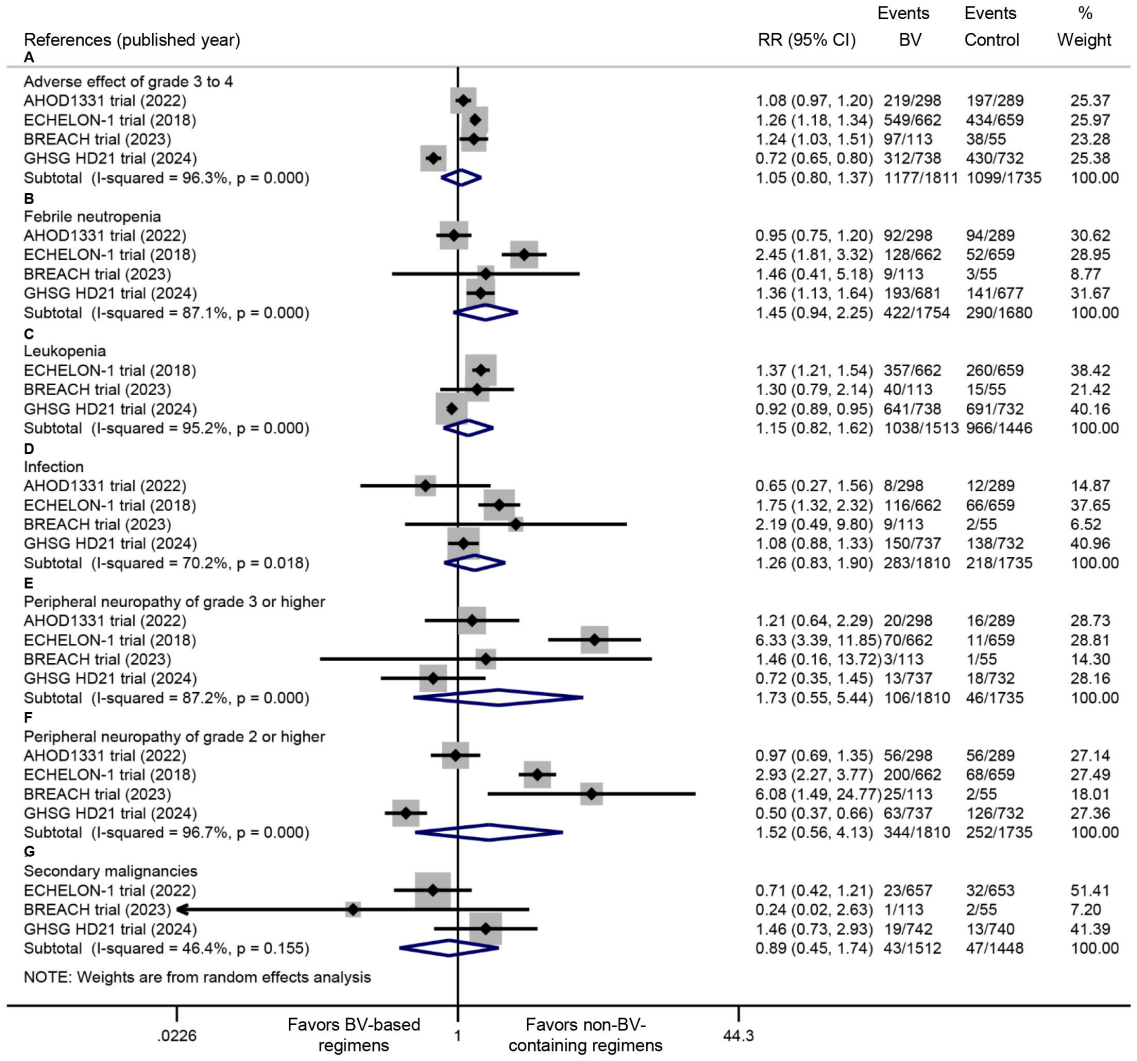
Comparative safety profiles of BV-based regimens versus conventional non-BV-containing regimens in newly diagnosed classical Hodgkin lymphoma, including **(A)** grade ≥3 adverse events, **(B)** febrile neutropenia, **(C)** leukopenia, **(D)** infections, **(E)** grade ≥3 peripheral neuropathy, **(F)** grade ≥2 peripheral neuropathy, and **(G)** secondary malignancies. BV: brentuximab vedotin. RR <1 favors BV-based regimens (safety outcomes).

All four RCTs documented the incidence of grade ≥3 AEs. Pooled analysis demonstrated comparable rates of grade ≥3 AEs between BV-based and conventional non-BV-containing regimens (RR: 1.05, 95% CI: 0.80 to 1.37, *P* = 0.739; *I^2^* = 96.3%, *P* < 0.001) (**Figure 5**). The safety profile evaluation for BV-based regimens specifically focused on febrile neutropenia, leukopenia, infections, peripheral neuropathy, and secondary malignancies. Meta-analysis of extracted trial data revealed no statistically significant differences in these predefined AEs between BV-based and conventional non-BV-containing regimens. Notably, while not reaching statistical significance, a trend toward higher febrile neutropenia incidence was observed with BV-based therapy (RR: 1.45, 95% CI: 0.94 to 2.25, *P* = 0.093; *I^2^* = 87.1%, *P* < 0.001) (**Figure 5**).

## 4 Discussion

To our knowledge, this is the first meta-analysis comprehensively comparing BV-based versus conventional non-BV-containing regimens in newly diagnosed classical HL using pooled data from all available RCTs. Our findings provide three key advances to the field: First, BV-based regimens demonstrated significant PFS improvements, with consistent benefits across clinically relevant subgroups. Second, BV’s therapeutic effects accumulate during treatment, evidenced by significantly superior end-of-treatment complete metabolic response rates despite comparable interim PET-2 negativity rates. Third, comprehensive safety evaluations confirmed that with appropriate granulocyte colony-stimulating factor (G-CSF) prophylaxis, BV-based regimens achieve comparable toxicity profiles to conventional non-BV-containing regimens.

Interim PET-2 status has established predictive value in HL, where early metabolic response correlates with superior disease control (20, 27, 28). Our meta-analysis revealed that BV-based regimens showed a clear trend toward higher PET-2 negative rates, and significantly improved end-of-treatment complete metabolic response rates. The superior PFS outcomes with BV-based regimens appear driven predominantly by the significantly enhanced complete metabolic responses at treatment completion, while the contribution of early PET-2 response remains uncertain given its non-significant difference (*P* = 0.286).

Our meta-analysis could not formally assess overall survival (OS) due to limited data and heterogeneous follow-up durations. In ECHELON-1 (73-month follow-up), BV-AVD demonstrated both significant OS benefit (HR: 0.59; 95% CI: 0.40 to 0.88) and lower mortality (5.9% vs ABVD’s 9.7%), with deaths primarily from disease progression or treatment complications (18). Other trials (BREACH [45 months], AHOD1331 [42 months], GHSG HD21 [48 months]) reported only 24 total deaths during shorter follow-up, reflecting their immature survival data. Although PFS serves as a validated surrogate for OS in HL (29) and the observed PFS advantage suggests potential survival benefit, the low mortality events in non-ECHELON trials preclude definitive conclusions. These findings highlight the need for extended follow-up to determine whether the PFS benefits with BV-based regimens consistently translate into OS advantages across all study populations.

Previous studies have reported increased febrile neutropenia (FN) with BV-based regimens (17, 20). However, our pooled analysis found no significant difference in FN rates, which was mainly attributed to the mandatory prophylactic use of G-CSF (19, 21). This finding was supported by the ECHELON-1 study, which confirmed that G-CSF prophylaxis reduced the risk of FN in the BV group to a level comparable with the control group (30). Therefore, G-CSF prophylaxis is strongly recommended when BV is used. Consistently, we observed comparable rates of leukopenia and infections between BV-based and conventional non-BV-containing regimens.

Peripheral neuropathy (PN) is a frequently reported AE associated with BV-based regimens, with grade ≥2 PN significantly impairing patients’ quality of life (31, 32). Our meta-analysis found no statistically significant increase in PN with BV-based regimens, with most cases either completely resolving or improving with longer follow-up; the majority of residual cases were grade 1 PN (18, 19, 33, 34). Regarding other severe toxicities, specifically the risk of secondary malignancies, our analysis detected no increased risk of secondary malignancies with BV-based regimens. However, these findings require cautious interpretation given the relatively short median follow-up.

Several limitations of this meta-analysis should be considered. First, this meta-analysis included four open-label trials lacking allocation concealment, potentially introducing bias in subjective endpoints: PET interpretation (despite Deauville criteria standardization) and toxicity assessments, particularly neurotoxicity. While objective PFS outcomes remain robust, these limitations necessitate cautious interpretation of metabolic response and safety data. Second, clinical heterogeneity existed regarding patient characteristics, BV treatment protocols, conventional non-BV-containing regimens, and follow-up duration across the included studies, which may have been responsible for the inconsistent results. Although sensitivity and subgroup analyses demonstrated consistent benefits with BV-based regimens, biological differences and protocol variations (e.g., treatment protocols, dose intensity, and drug tolerance) between pediatric and adult populations warrant caution when generalizing these effects. Finally, we exclusively compared BV-based versus conventional non-BV-containing regimens in this meta-analysis; the comparisons between BV with other new agents, such as immune checkpoint inhibitors (35), in newly diagnosed classical HL patients need to be further investigated.

In summary, our meta-analysis demonstrates that BV-based regimens offer meaningful PFS benefits with manageable toxicity profiles in newly diagnosed classical HL patients. These findings strongly support the incorporation of BV into frontline treatment strategies for classical HL.

## Data Availability

The original contributions presented in the study are included in the article/**Supplementary Material**. Further inquiries can be directed to the corresponding authors.

## References

[1] Duggan DB Petroni GR Johnson JL Glick JH Fisher RI Connors JM. Randomized comparison of ABVD and MOPP/ABV hybrid for the treatment of advanced Hodgkin’s disease: report of an intergroup trial. J Clin Oncol. (2003) 21:607–14. doi: 10.1200/jco.2003.12.086, PMID: 12586796

[2] Meyer RM Gospodarowicz MK Connors JM Pearcey RG Wells WA Winter JN. ABVD alone versus radiation-based therapy in limited-stage Hodgkin’s lymphoma. N Engl J Med. (2012) 366:399–408. doi: 10.1056/NEJMoa1111961, PMID: 22149921 PMC3932020

[3] Carde P Karrasch M Fortpied C Brice P Khaled H Casasnovas O. Eight cycles of ABVD versus four cycles of BEACOPPescalated plus four cycles of BEACOPPbaseline in stage III to IV, international prognostic score ≥ 3, high-risk hodgkin lymphoma: first results of the phase III EORTC 20012 intergroup trial. J Clin Oncol. (2016) 34:2028–36. doi: 10.1200/jco.2015.64.5648, PMID: 27114593

[4] Gordon LI Hong F Fisher RI Bartlett NL Connors JM Gascoyne RD. Randomized phase III trial of ABVD versus Stanford V with or without radiation therapy in locally extensive and advanced-stage Hodgkin lymphoma: an intergroup study coordinated by the Eastern Cooperative Oncology Group (E2496). J Clin Oncol. (2013) 31:684–91. doi: 10.1200/jco.2012.43.4803, PMID: 23182987 PMC3574266

[5] Viviani S Zinzani PL Rambaldi A Brusamolino E Levis A Bonfante V. ABVD versus BEACOPP for Hodgkin’s lymphoma when high-dose salvage is planned. N Engl J Med. (2011) 365:203–12. doi: 10.1056/NEJMoa1100340, PMID: 21774708

[6] Eichenauer DA Becker I Monsef I Chadwick N de Sanctis V Federico M. Secondary Malignant neoplasms, progression-free survival and overall survival in patients treated for Hodgkin lymphoma: a systematic review and meta-analysis of randomized clinical trials. Haematologica. (2017) 102:1748–57. doi: 10.3324/haematol.2017.167478, PMID: 28912173 PMC5622859

[7] Stephens DM Li H Schöder H Straus DJ Moskowitz CH LeBlanc M. Five-year follow-up of SWOG S0816: limitations and values of a PET-adapted approach with stage III/IV Hodgkin lymphoma. Blood. (2019) 134:1238–46. doi: 10.1182/blood.2019000719, PMID: 31331918 PMC6788007

[8] André MPE Carde P Viviani S Bellei M Fortpied C Hutchings M. Long-term overall survival and toxicities of ABVD vs BEACOPP in advanced Hodgkin lymphoma: A pooled analysis of four randomized trials. Cancer Med. (2020) 9:6565–75. doi: 10.1002/cam4.3298, PMID: 32710498 PMC7520354

[9] Katz J Janik JE Younes A. Brentuximab vedotin (SGN-35). Clin Cancer Res. (2011) 17:6428–36. doi: 10.1158/1078-0432.Ccr-11-0488, PMID: 22003070

[10] Younes A Gopal AK Smith SE Ansell SM Rosenblatt JD Savage KJ. Results of a pivotal phase II study of brentuximab vedotin for patients with relapsed or refractory Hodgkin’s lymphoma. J Clin Oncol. (2012) 30:2183–9. doi: 10.1200/jco.2011.38.0410, PMID: 22454421 PMC3646316

[11] Chen R Gopal AK Smith SE Ansell SM Rosenblatt JD Savage KJ. Five-year survival and durability results of brentuximab vedotin in patients with relapsed or refractory Hodgkin lymphoma. Blood. (2016) 128:1562–6. doi: 10.1182/blood-2016-02-699850, PMID: 27432875 PMC5034737

[12] Moskowitz CH Nademanee A Masszi T Agura E Holowiecki J Abidi MH. Brentuximab vedotin as consolidation therapy after autologous stem-cell transplantation in patients with Hodgkin’s lymphoma at risk of relapse or progression (AETHERA): a randomised, double-blind, placebo-controlled, phase 3 trial. Lancet. (2015) 385:1853–62. doi: 10.1016/s0140-6736(15)60165-9, PMID: 25796459

[13] Evens AM Advani RH Helenowski IB Fanale M Smith SM Jovanovic BD. Multicenter phase II study of sequential brentuximab vedotin and doxorubicin, vinblastine, and dacarbazine chemotherapy for older patients with untreated classical hodgkin lymphoma. J Clin Oncol. (2018) 36:3015–22. doi: 10.1200/jco.2018.79.0139, PMID: 30179569

[14] O’Connor OA Lue JK Sawas A Amengual JE Deng C Kalac M. Brentuximab vedotin plus bendamustine in relapsed or refractory Hodgkin’s lymphoma: an international, multicentre, single-arm, phase 1–2 trial. Lancet Oncol. (2018) 19:257–66. doi: 10.1016/s1470-2045(17)30912-9, PMID: 29276022 PMC9098158

[15] Rubinstein PG Moore PC Bimali M Lee JY Rudek MA Chadburn A. Brentuximab vedotin with AVD for stage II-IV HIV-related Hodgkin lymphoma (AMC 085): phase 2 results from an open-label, single arm, multicentre phase 1/2 trial. Lancet Haematol. (2023) 10:e624–e32. doi: 10.1016/s2352-3026(23)00157-6, PMID: 37532416 PMC10859222

[16] Forlenza CJ Gulati N Mauguen A Absalon MJ Castellino SM Franklin A. Combination brentuximab vedotin and bendamustine for pediatric patients with relapsed/refractory Hodgkin lymphoma. Blood Adv. (2021) 5:5519–24. doi: 10.1182/bloodadvances.2021005268, PMID: 34559223 PMC8714712

[17] Connors JM Jurczak W Straus DJ Ansell SM Kim WS Gallamini A. Brentuximab vedotin with chemotherapy for stage III or IV hodgkin’s lymphoma. N Engl J Med. (2018) 378:331–44. doi: 10.1056/NEJMoa1708984, PMID: 29224502 PMC5819601

[18] Ansell SM Radford J Connors JM Długosz-Danecka M Kim WS Gallamini A. Overall survival with brentuximab vedotin in stage III or IV hodgkin’s lymphoma. N Engl J Med. (2022) 387:310–20. doi: 10.1056/NEJMoa2206125, PMID: 35830649

[19] Borchmann P Ferdinandus J Schneider G Moccia A Greil R Hertzberg M. Assessing the efficacy and tolerability of PET-guided BrECADD versus eBEACOPP in advanced-stage, classical Hodgkin lymphoma (HD21): a randomised, multicentre, parallel, open-label, phase 3 trial. Lancet. (2024) 404:341–52. doi: 10.1016/s0140-6736(24)01315-1, PMID: 38971175

[20] Fornecker LM Lazarovici J Aurer I Casasnovas RO Gac AC Bonnet C. Brentuximab vedotin plus AVD for first-line treatment of early-stage unfavorable hodgkin lymphoma (BREACH): A multicenter, open-label, randomized, phase II trial. J Clin Oncol. (2023) 41:327–35. doi: 10.1200/jco.21.01281, PMID: 35867960

[21] Castellino SM Pei Q Parsons SK Hodgson D McCarten K Horton T. Brentuximab vedotin with chemotherapy in pediatric high-risk hodgkin’s lymphoma. N Engl J Med. (2022) 387:1649–60. doi: 10.1056/NEJMoa2206660, PMID: 36322844 PMC9945772

[22] Page MJ McKenzie JE Bossuyt PM Boutron I Hoffmann TC Mulrow CD. The PRISMA 2020 statement: an updated guideline for reporting systematic reviews. Bmj. (2021) 372:n71. doi: 10.1136/bmj.n71, PMID: 33782057 PMC8005924

[23] Cheson BD Fisher RI Barrington SF Cavalli F Schwartz LH Zucca E. Recommendations for initial evaluation, staging, and response assessment of Hodgkin and non-Hodgkin lymphoma: the Lugano classification. J Clin Oncol. (2014) 32:3059–68. doi: 10.1200/jco.2013.54.8800, PMID: 25113753 PMC4979083

[24] Higgins JP Altman DG Gotzsche PC Juni P Moher D Oxman AD. The Cochrane Collaboration’s tool for assessing risk of bias in randomised trials. Bmj. (2011) 343:d5928. doi: 10.1136/bmj.d5928, PMID: 22008217 PMC3196245

[25] Higgins JP Thompson SG. Quantifying heterogeneity in a meta-analysis. Stat Med. (2002) 21:1539–58. doi: 10.1002/sim.1186, PMID: 12111919

[26] DerSimonian R Laird N. Meta-analysis in clinical trials. Control Clin Trials. (1986) 7:177–88. doi: 10.1016/0197-2456(86)90046-2, PMID: 3802833

[27] Johnson P Federico M Kirkwood A Fosså A Berkahn L Carella A. Adapted treatment guided by interim PET-CT scan in advanced hodgkin’s lymphoma. N Engl J Med. (2016) 374:2419–29. doi: 10.1056/NEJMoa1510093, PMID: 27332902 PMC4961236

[28] Hutchings M Loft A Hansen M Pedersen LM Buhl T Jurlander J. FDG-PET after two cycles of chemotherapy predicts treatment failure and progression-free survival in Hodgkin lymphoma. Blood. (2006) 107:52–9. doi: 10.1182/blood-2005-06-2252, PMID: 16150944

[29] Bröckelmann PJ Müller H Fuchs M Gillessen S Eichenauer DA Borchmann S. Correlation between progression-free and overall survival in patients with Hodgkin lymphoma: a comprehensive analysis of individual patient data from randomized German Hodgkin Study Group (GHSG) trials. Ann Oncol. (2025) 36:393–402. doi: 10.1016/j.annonc.2024.12.009, PMID: 39706337

[30] Straus D Collins G Walewski J Zinzani PL Grigg A Sureda A. Primary prophylaxis with G-CSF may improve outcomes in patients with newly diagnosed stage III/IV Hodgkin lymphoma treated with brentuximab vedotin plus chemotherapy. Leuk Lymphoma. (2020) 61:2931–8. doi: 10.1080/10428194.2020.1791846, PMID: 32842815

[31] Bowers JT Anna J Bair SM Annunzio K Epperla N Pullukkara JJ. Brentuximab vedotin plus AVD for Hodgkin lymphoma: incidence and management of peripheral neuropathy in a multisite cohort. Blood Adv. (2023) 7:6630–8. doi: 10.1182/bloodadvances.2023010622, PMID: 37595053 PMC10628810

[32] Zinzani PL Ramchandren R Santoro A Paszkiewicz-Kozik E Gasiorowski R Johnson NA. Quality-of-life analysis of pembrolizumab vs brentuximab vedotin for relapsed/refractory classical Hodgkin lymphoma. Blood Adv. (2022) 6:590–9. doi: 10.1182/bloodadvances.2021004970, PMID: 34644372 PMC8791579

[33] Straus DJ Długosz-Danecka M Alekseev S Illés Á Picardi M Lech-Maranda E. Brentuximab vedotin with chemotherapy for stage III/IV classical Hodgkin lymphoma: 3-year update of the ECHELON-1 study. Blood. (2020) 135:735–42. doi: 10.1182/blood.2019003127, PMID: 31945149

[34] Straus DJ Długosz-Danecka M Connors JM Alekseev S Illés Á Picardi M. Brentuximab vedotin with chemotherapy for stage III or IV classical Hodgkin lymphoma (ECHELON-1): 5-year update of an international, open-label, randomised, phase 3 trial. Lancet Haematol. (2021) 8:e410–e21. doi: 10.1016/s2352-3026(21)00102-2, PMID: 34048680

[35] Herrera AF LeBlanc M Castellino SM Li H Rutherford SC Evens AM. Nivolumab+AVD in advanced-stage classic hodgkin’s lymphoma. N Engl J Med. (2024) 391:1379–89. doi: 10.1056/NEJMoa2405888, PMID: 39413375 PMC11488644

