# Supplementary material for: Frontline brentuximab vedotin-based therapy for newly diagnosed classical Hodgkin lymphoma: a meta-analysis of randomized controlled trials
Source: Front Oncol. 2025 Jul 21;15:1636923. doi: 10.3389/fonc.2025.1636923 (PMC12318742; doi:10.3389/fonc.2025.1636923)
Supplement: Supplementary file 1 [file DataSheet1.docx]

**Table S1. Search criterion of PubMed (from inception to Mar 25, 2025)**

| **No.** | **Query Results** | **Results** |
| --- | --- | --- |
| #13 | #3 and #9 and #12 | 74 |
| #12 | #10 or #11 | 1125710 |
| #11 | Search: randomize*[Text Word] | 1124688 |
| #10 | Search: "Randomized Controlled Trial" [Publication Type] Sort by: Most Recent | 636595 |
| #9 | #4 or #5 or #6 or #7 or #8 | 76886 |
| #8 | Search: Hodgkin's disease[Title/Abstract] | 19836 |
| #7 | Search: Hodgkin disease[Title/Abstract] | 1740 |
| #6 | Search: Hodgkin’s lymphoma[Title/Abstract] | 25872 |
| #5 | Search: Hodgkin lymphoma[Title/Abstract] | 23630 |
| #4 | Search: "Hodgkin Disease"[Mesh] Sort by: Most Recent | 36416 |
| #3 | #1 or #2 | 1492 |
| #2 | Search: brentuximab vedotin[Title/Abstract] | 1491 |
| #1 | Search: "Brentuximab Vedotin"[Mesh] Sort by: Most Recent | 955 |

**Table S2. Search criterion of Embase (from inception to Mar 25, 2025)**

| **No.** | **Query Results** | **Results** |
| --- | --- | --- |
| #11 | #3 and #7 and #10 | 539 |
| #10 | #8 or #9 | 2492824 |
| #9 | 'randomized controlled trial'/exp | 879335 |
| #8 | random* | 2491271 |
| #7 | #4 or #5 or #6 | 143562 |
| #6 | 'hodgkin disease'/exp | 72540 |
| #5 | hodgkin AND lymphoma:ti,ab,kw | 100530 |
| #4 | hodgkin AND disease:ti,ab,kw | 67584 |
| #3 | #1 or #2 | 6838 |
| #2 | 'brentuximab vedotin'/exp | 6613 |
| #1 | brentuximab AND vedotin:ti,ab,kw | 3547 |

**Table S3. Search criterion of Cochrane Library (from inception to Mar 25, 2025)**

| **No.** | **Query Results** | **Results** |
| --- | --- | --- |
| #1 | (Brentuximab Vedotin):ti,ab,kw | 409 |
| #2 | MeSH descriptor: [Brentuximab Vedotin] explode all trees | 86 |
| #3 | #1 or #2 | 409 |
| #4 | (Hodgkin's disease):ti,ab,kw | 4138 |
| #5 | (Hodgkin disease):ti,ab,kw | 4138 |
| #6 | (Hodgkin’s lymphoma):ti,ab,kw | 5515 |
| #7 | (Hodgkin lymphoma):ti,ab,kw | 5515 |
| #8 | MeSH descriptor: [Hodgkin Disease] explode all trees | 1048 |
| #9 | #4 or #5 or #6 or #7 or #8 | 6421 |
| #10 | MeSH descriptor: [Randomized Controlled Trial] explode all trees | 34 |
| #11 | (random*):ti,ab,kw | 1380317 |
| #12 | #10 or #11 | 1380317 |
| #13 | #3 and #9 and #12 | 209 |

**Table S4. Characteristics of excluded full-text studies**

| **Study** | **Reason for exclusion** |
| --- | --- |
| Herrera et al ^1^ | Other comparison: BV-AVD vs. Nivolumab-AVD |
| Kuruvilla et al ^2^ | Other comparison: BV-AVD vs. Pembrolizumab-AVD |
| Damaschin et al ^3^ | Other comparison: BrECAPP vs. BrECADD |
| Eichenauer et al ^4^ | Other comparison: BrECAPP vs. BrECADD |
| Connors et al ^5^ | Other comparison: BV-ABVD vs. BV-AVD |
| Huntington et al ^6^ | Other outcomes: cost-effectiveness |
| Delea et al ^7^ | Other outcomes: cost-effectiveness |
| Hui et al ^8^ | Other outcomes: cost-effectiveness |
| Williams et al ^9^ | Other outcomes: health related quality of life |
| Moskowitz et al ^10^ | Other patients: patients with HL after transplantation |
| Moskowitz et al ^11^ | Other patients: patients with HL after transplantation |
| Ramsey et al ^12^ | Other patients: patients with HL after transplantation |
| Ramchandren et al ^13^ | Other reasons: subgroup analysis of ECHELON-1 trial |
| Zhang et al ^14^ | Other reasons: exposure-response of AHOD1331 trial |

Abbreviations: BV: brentuximab vedotin; AVD: doxorubicin, vinblastine, and dacarbazine; BrECAPP: brentuximab vedotin, etoposide, cyclophosphamide, doxorubicin, procarbazine, and prednisone; BrECADD: brentuximab vedotin, etoposide, cyclophosphamide, doxorubicin, dacarbazine, and dexamethasone.

**References**

**1.** Herrera AF, LeBlanc M, Castellino SM, et al. Nivolumab+AVD in Advanced-Stage Classic Hodgkin's Lymphoma. *N Engl J Med.* 2024;391 (15):1379-1389. 10.1056/NEJMoa2405888.

**2.** Kuruvilla J, Ramchandren R, Santoro A, et al. Pembrolizumab versus brentuximab vedotin in relapsed or refractory classical Hodgkin lymphoma (KEYNOTE-204): an interim analysis of a multicentre, randomised, open-label, phase 3 study. *Lancet Oncol.* 2021;22 (4):512-524. doi:10.1016/s1470-2045(21)00005-x.

**3.** Damaschin C, Goergen H, Kreissl S, et al. Brentuximab vedotin-containing escalated BEACOPP variants for newly diagnosed advanced-stage classical Hodgkin lymphoma: follow-up analysis of a randomized phase II study from the German Hodgkin Study Group. *Leukemia.* 2022;36 (2):580-582. doi:10.1038/s41375-021-01386-z.

**4.** Eichenauer DA, Plütschow A, Kreissl S, et al. Incorporation of brentuximab vedotin into first-line treatment of advanced classical Hodgkin's lymphoma: final analysis of a phase 2 randomised trial by the German Hodgkin Study Group. *Lancet Oncol.* 2017;18 (12):1680-1687. doi:10.1016/s1470-2045(17)30696-4.

**5.** Connors JM, Ansell SM, Fanale M, Park SI, Younes A. Five-year follow-up of brentuximab vedotin combined with ABVD or AVD for advanced-stage classical Hodgkin lymphoma. *Blood.* 2017;130 (11):1375-1377. doi:10.1182/blood-2017-05-784678.

**6.** Huntington SF, von Keudell G, Davidoff AJ, Gross CP, Prasad SA. Cost-Effectiveness Analysis of Brentuximab Vedotin With Chemotherapy in Newly Diagnosed Stage III and IV Hodgkin Lymphoma. *J Clin Oncol.* 2018;36 (33):Jco1800122. doi:10.1200/jco.18.00122.

**7.** Delea TE, Sharma A, Grossman A, et al. Cost-effectiveness of brentuximab vedotin plus chemotherapy as frontline treatment of stage III or IV classical Hodgkin lymphoma. *J Med Econ.* 2019;22 (2):117-130. doi:10.1080/13696998.2018.1542599.

**8.** Hui L, von Keudell G, Wang R, et al. Cost-effectiveness analysis of consolidation with brentuximab vedotin for high-risk Hodgkin lymphoma after autologous stem cell transplantation. *Cancer.* 2017;123 (19):3763-3771. doi:10.1002/cncr.30818.

**9.** Williams AM, Rodday AM, Pei Q, et al. Longitudinal Health-Related Quality of Life Among Patients With High-Risk Pediatric Hodgkin Lymphoma Treated on the Children's Oncology Group AHOD 1331 Study. *J Clin Oncol.* 2024;42 (28):3330-3338. doi:10.1200/jco.24.00038.

**10.** Moskowitz CH, Nademanee A, Masszi T, et al. Brentuximab vedotin as consolidation therapy after autologous stem-cell transplantation in patients with Hodgkin's lymphoma at risk of relapse or progression (AETHERA): a randomised, double-blind, placebo-controlled, phase 3 trial. *Lancet.* 2015;385 (9980):1853-1862. doi:10.1016/s0140-6736(15)60165-9.

**11.** Moskowitz CH, Walewski J, Nademanee A, et al. Five-year PFS from the AETHERA trial of brentuximab vedotin for Hodgkin lymphoma at high risk of progression or relapse. *Blood.* 2018;132 (25):2639-2642. doi:10.1182/blood-2018-07-861641.

**12.** Ramsey SD, Nademanee A, Masszi T, et al. Quality of life results from a phase 3 study of brentuximab vedotin consolidation following autologous haematopoietic stem cell transplant for persons with Hodgkin lymphoma. *Br J Haematol.* 2016;175 (5):860-867. doi:10.1111/bjh.14316.

**13.** Ramchandren R, Advani RH, Ansell SM, et al. Brentuximab Vedotin plus Chemotherapy in North American Subjects with Newly Diagnosed Stage III or IV Hodgkin Lymphoma. *Clin Cancer Res.* 2019;25 (6):1718-1726. doi:10.1158/1078-0432.Ccr-18-2435.

**14.** Zhang Z, Zhang D, Guo W, et al. Exposure-Response and Subgroup Analyses to Support Body Weight-Based Dosing of Brentuximab Vedotin in Children and Young Adults with Newly Diagnosed High-risk Classical Hodgkin Lymphoma. *Clin Cancer Res.* 2024;30 (15):3273-3281. doi:10.1158/1078-0432.Ccr-23-3655.

**
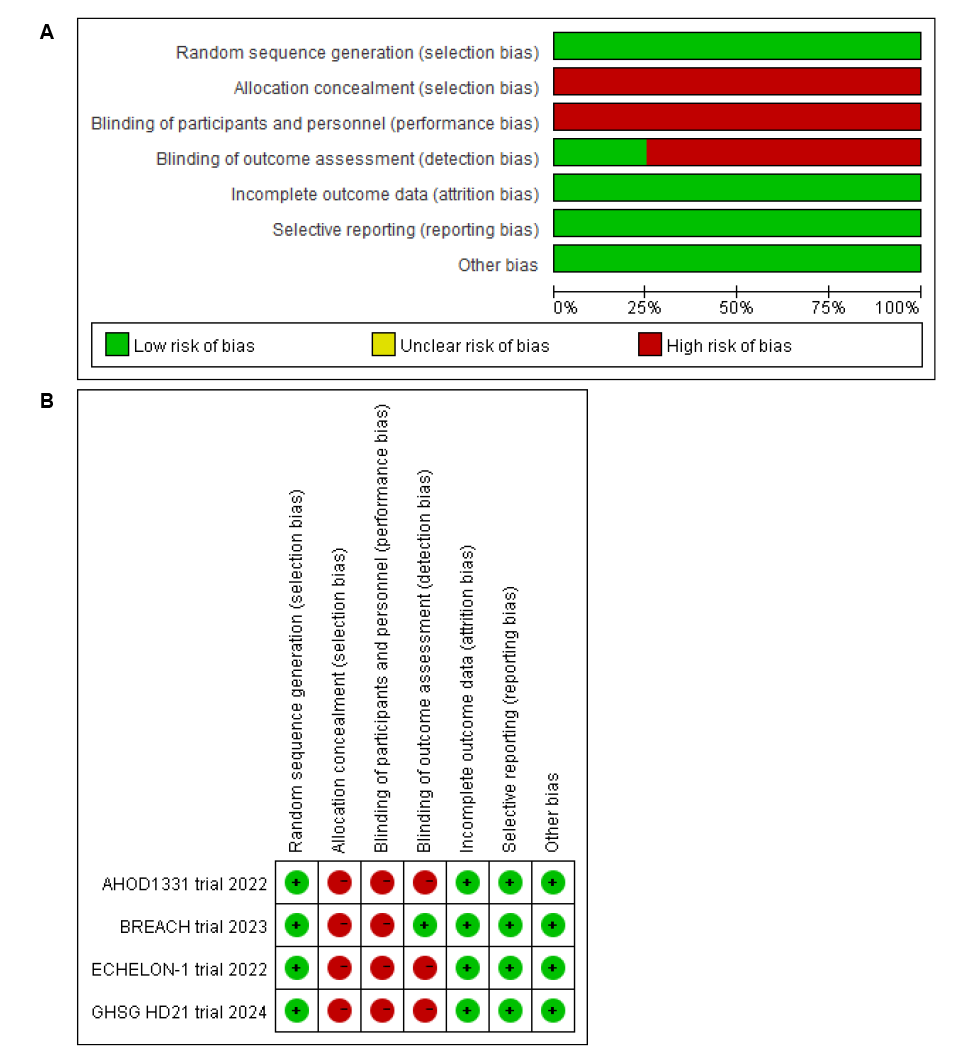
**

**Figure S1.** Methodological quality of the included studies. (**A**) Risk of bias graph. (**B**) Risk of bias summary. The minus sign indicates a high risk of bias, and the plus sign indicates a low risk of bias.

**Table S5. Sensitivity analysis of PFS data**

| Study Omitted | Meta-analysis |
| --- | --- |
| AHOD1331 trial | HR: 0.66, 95% CI: 0.54 to 0.81 |
| ECHELON-1 trial | HR: 0.50, 95% CI: 0.33 to 0.77 |
| BREACH trial | HR: 0.60, 95% CI: 0.46 to 0.79 |
| GHSG HD21 trial | HR: 0.51, 95% CI: 0.32 to 0.82 |

HR <1 favors BV-based regimens.
